# Supplementary material for: The New Buffer Salt-Protected Sodium Butyrate Promotes Growth Performance by Improving Intestinal Histomorphology, Barrier Function, Antioxidative Capacity, and Microbiota Community of Broilers
Source: Biology (Basel). 2024 May 1;13(5):317. doi: 10.3390/biology13050317 (PMC11117952; doi:10.3390/biology13050317)
Supplement: Supplementary file 1 [file biology-13-00317-s001.zip › biology-2961844-supplementary.pdf]

Supplementary Table S1. Primers used for real-time quantitative PCR (RT-qPCR) analysis.

Gene	Forward primer (5′ s) and Reverse primer (3′s)	GenBank Accession No.	Bp (product size)
ZO-1	F: AATACCTGACTGTCTTGCAG	XM_015278975.1	145
	R: TAAAGAAGGCTTTCCCTGAC		
Claudin-1	F: CAGACTCTAGGTTTTGCCTT	NM_001013611.2	149
	R: AATCTTTCCAGTGGCGATAC		
Occludin	F: TCGTGCTGTGCATCGCCATC	NM_205128.1	178
	R: CGCTGGTTCACCCCTCCGTA		
Mucin-2	F: CTGATTGTCACTCACGCCTTAATC	XM_001234581.3	147
	R: GCCGGCCACCTGCAT		
β-actin	F: ACCTGAGCGCAAGTACTCTGTCT	NM_205518.1	95
	R: CATCGTACTCCTGCTTGCTGAT		
